# Home Care Nurses’ Experiences of the Use and Introduction of the Subacute Functional Decline in the Elderly Instrument

**DOI:** 10.1177/23779608231187246

**Published:** 2023-08-10

**Authors:** Nima Wesseltoft-Rao, Zada Pajalic, Annabel Hamre, Abdallah Abudayya

**Affiliations:** 1 Faculty of Health Sciences, 87368 VID Specialized University, Oslo, Norway

**Keywords:** nursing assessment, assessment of healthcare needs, home care service, observations, aged

## Abstract

**Introduction:**

Registered nurses are crucial in home care nursing for elderly patients, as detecting geriatric conditions can be difficult due to age-related changes or communication barriers. Disability is often overlooked in elderly care, requiring different assessment tools to determine patient status and necessary nursing interventions. During the COVID-19 pandemic, the subacute functional decline in the elderly (SAFE) instrument was implemented in some Oslo districts to detect early signs of sub acute functional decline in hospital and home care settings. However, the nurses’ perception of this new assessment tool and its effectiveness has not been evaluated.

**Objectives:**

This study aims to explore home care nurses’ experiences and perceptions regarding the introduction and use of the new assessment tool, SAFE. Objectives were to conduct focus group interviews and perform qualitative analysis.

**Method:**

The study followed Consolidated Criteria for Reporting Qualitative Research guidelines, had a qualitative design, and included 15 out of 60 permanently employed RNs at Oslo municipality's home care service in Frogner district. Data was collected via three focus group interviews and analyzed thematically.

**Results:**

The study identified three themes: (1) Nurses learned to use SAFE through direct experience due to a lack of standard introduction or training. (2) SAFE supported patient-centred care by enabling communication, preventive work, and identifying patients’ needs. (3) Integrating SAFE into electronic databases and daily clinical work could improve nursing efficiency.

**Conclusion:**

Overall, using SAFE can improve patient outcomes and care quality in home care, but clear guidelines, ongoing support, and standardized procedures are crucial for its effectiveness. Regular updates and complete management support are also necessary. The study's findings align with previous research and can guide the development and implementation of tools in home care to enhance patient outcomes and the quality of care delivered.

## Introduction

The quality of care for older adults living in their own homes depends on the quality of care provided by home care nurses. The use of various screening tools to systematically observe the status of a function is a prerequisite for providing optimal care (Landi et al., 2000). Registered nurses (RNs) play a crucial role in home care settings. Home care requires highly qualified nurses with different abilities, unique characteristics, experience-based knowledge and commitment to be able to coordinate care plans in the home. Home care is provided in the care receivers’ homes. Home care nursing requires continuous training for nurses to be able to adapt to complex and often unpredictable conditions (Andrade et al., 2017). In addition to licensed nurses, home care involves several health professionals intending to offer the best possible care in the home. Interprofessional collaboration and sharing knowledge and experiences are crucial for maintaining a high level of care in private homes (Pajalic, 2014). It can be challenging to detect geriatric conditions due to the ageing process, multimorbidity, cognitive impairment and difficulty communicating (Seematter-Bagnoud & Büla, 2018). The World Health Organization (WHO) recommends implementing screening to detect conditions that will require treatment (World Health Organization, 2017). The WHO's recommendations highlight the need to assess impairment of mental and physical functions (mobility, mood, vision, hearing, nutrition, cognitive risk of falls, incontinence, and depression). Disability is often overlooked in the care of the elderly. Therefore, there is a need to use different tests and assessment tools to assess patient status, prognosis and the need for different nursing interventions (Seematter-Bagnoud & Büla, 2018; World Health Organization, 2017). In the report “Diagnostic accuracy of a screening tool for non-specialist health care settings,” WHO has listed several screening tools.

## Review of Literature

**Figure 1.**
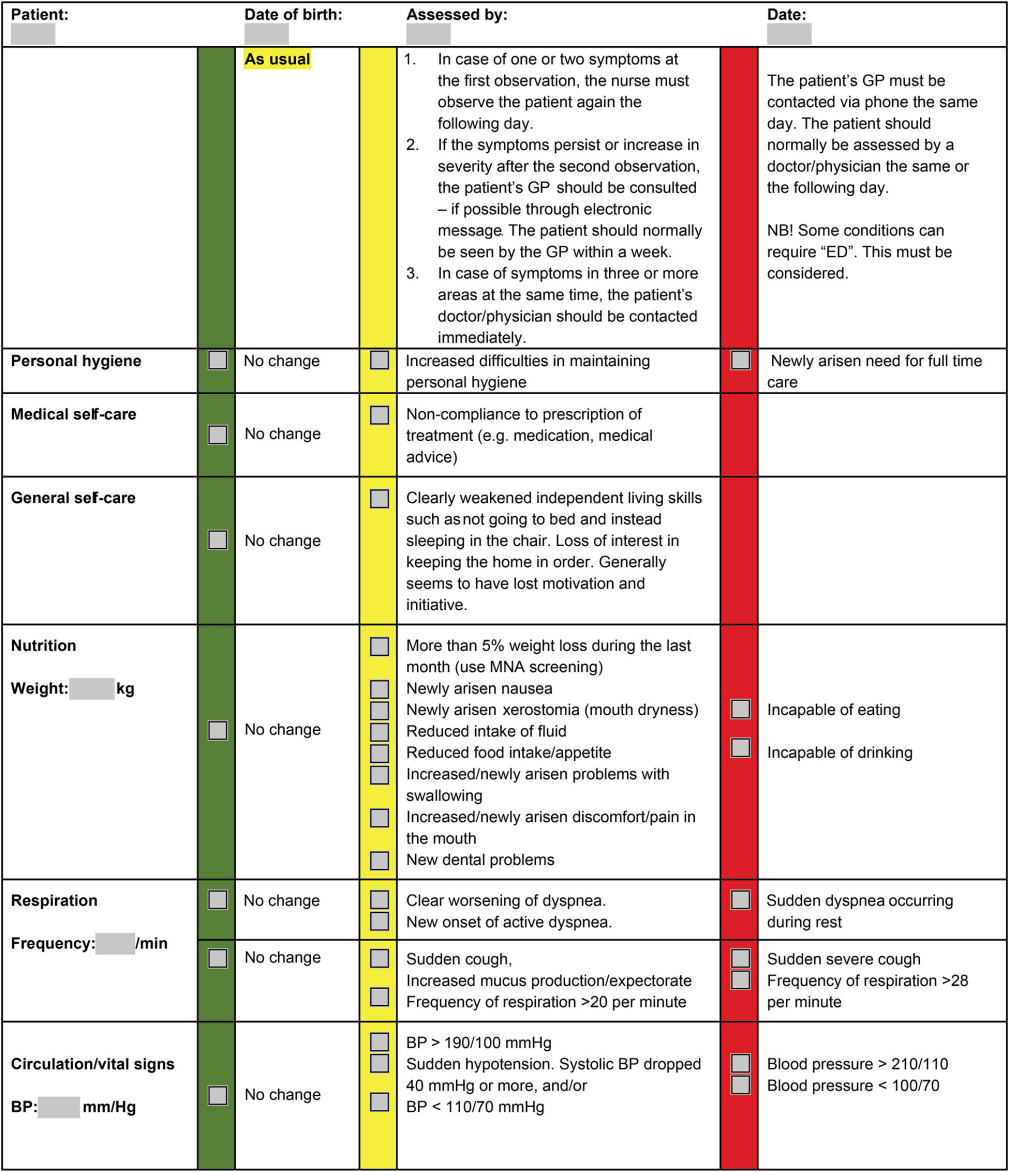

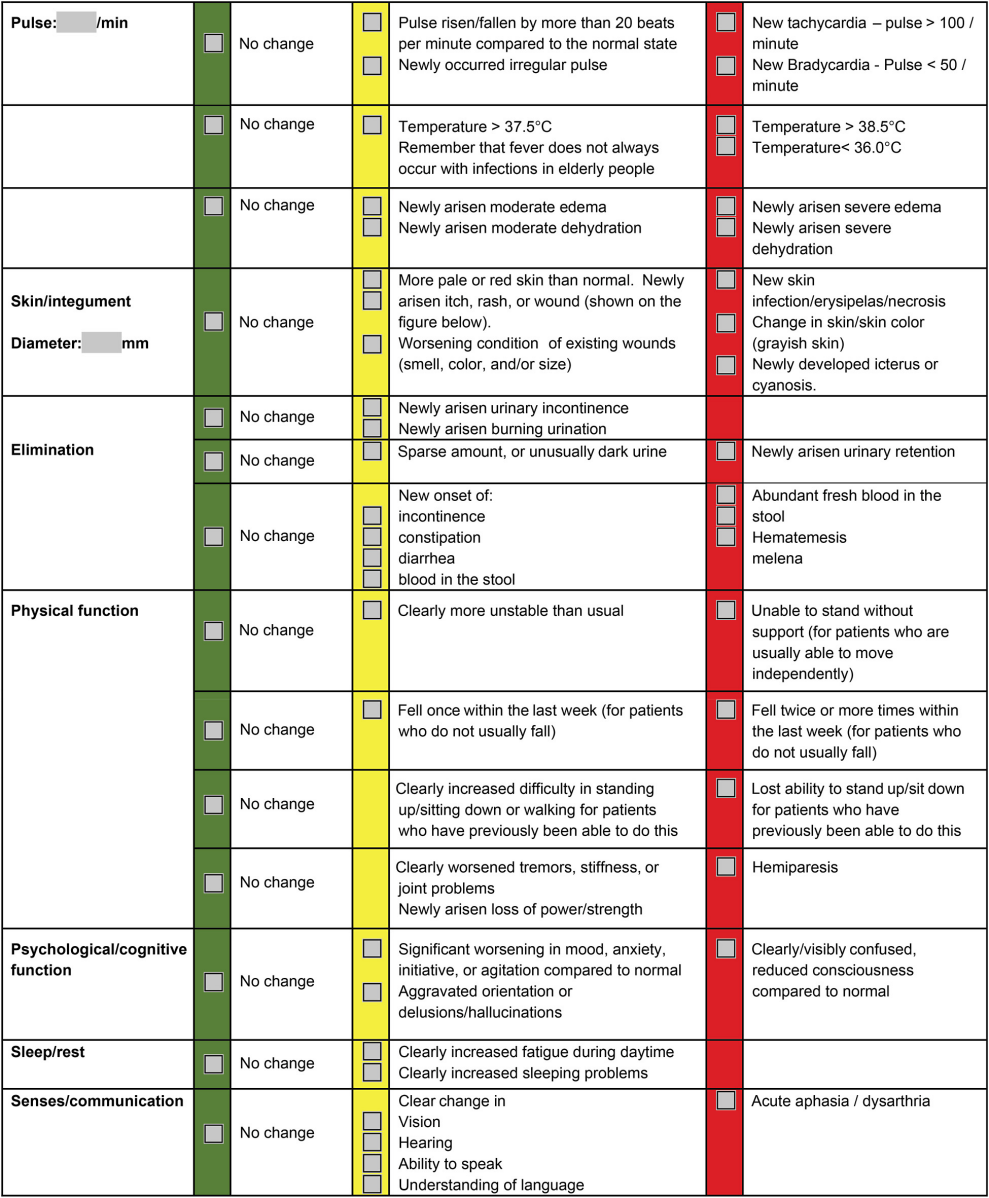

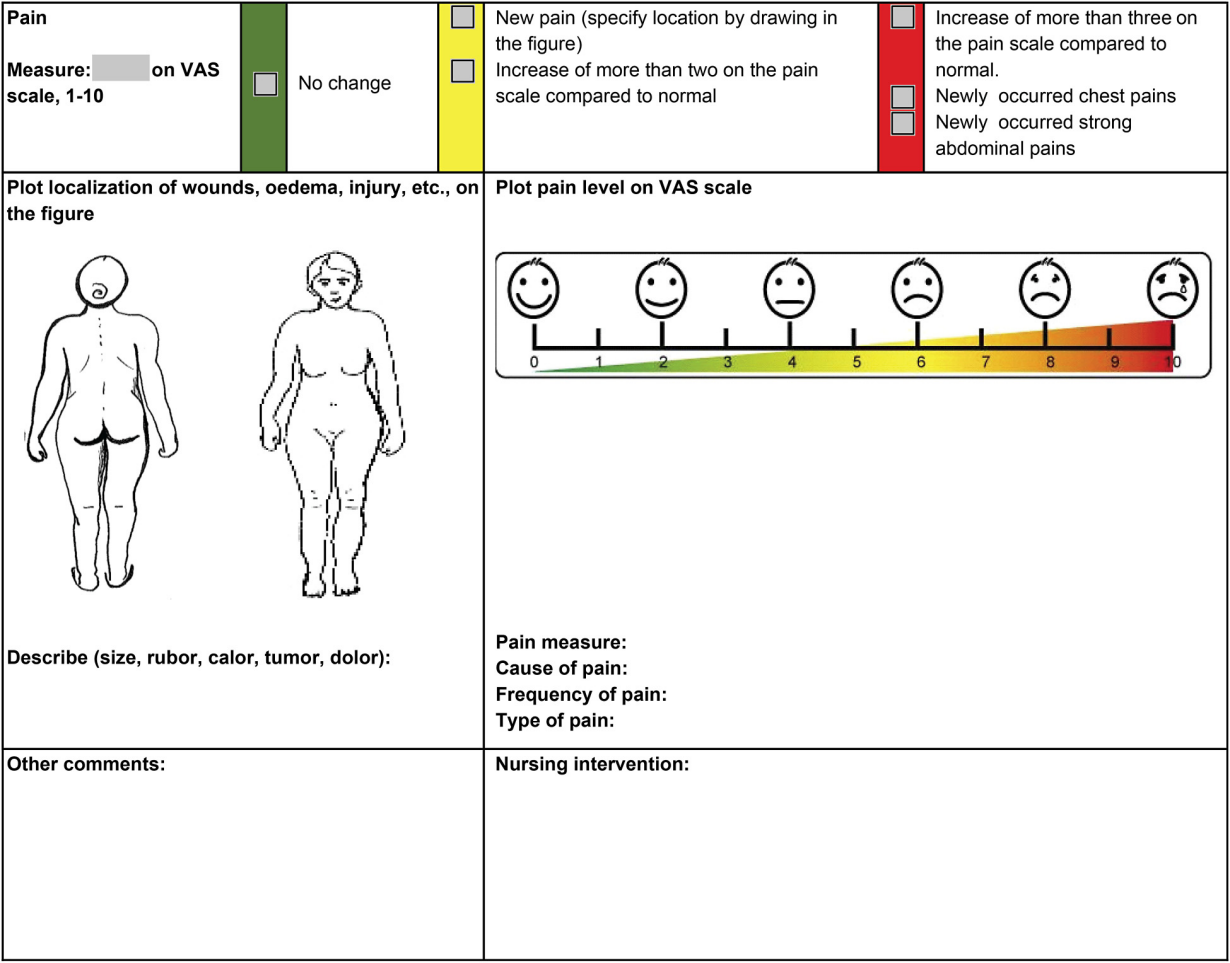
Acute functional decline in the elderly (SAFE) instrument.

The subacute functional decline in the elderly (SAFE) instrument is an assessment tool that was developed, evaluated and revised as part of a collaborative project between Oslo University Hospital and three districts in Oslo municipality. SAFE is intended to be used to detect early signs of sub acute and acute functional decline in older people who are cared for in hospitals and municipal home health care. Gjevjon et al. (2019) described SAFE as a structured observation and evaluation system used in home nursing care to detect changes in patient health status (Gjevjon et al., 2019). It consists of 13 main areas of observation and evaluation, and nurses use it to become familiar with a patient's normal state and to detect changes easily. The system uses yellow and red categories to indicate sub-acute and acute changes, respectively, while green indicates no change (Figure 1). Nurses use their professional judgment to determine whether there is a change based on the patient's normal state and previous SAFE status for comparison and follow-up. SAFE was implemented in some districts in Oslo during the COVID-19 pandemic, a time with increased needs to detect functional decline in users of home care services and is perceived an essential tool in home care nursing (Gjevjon et al., 2013, 2019; Gjevjon & Hellesø, 2010). Recent studies indicate that SAFE can enhance a structured follow-up of frail older people (Naess et al., 2017, 2023). Identification of the challenges nurses are facing in the process of implementing a new assessment tool, such as SAFE, may ensure the correct use of the tool (Abudayya et al., 2023). However, the use of SAFE and the nurse's perception of the tool has not been addressed. This study aims to explore home care nurses’ experiences and perceptions regarding the introduction and use of the new assessment tool, SAFE. Objectives are to conduct focus group (FG) interviews and perform qualitative thematic analysis.

## Method

### The Context of the Study

In Norway, the municipal healthcare system provides home care nursing services to individuals residing in their homes. These services are primarily delivered by RNs who conduct home visits and are responsible for monitoring the patient's general health condition and response to medication. If necessary, the RN collaborates with other healthcare professionals, such as general practitioners, physiotherapists, and occupational therapists, to ensure the best possible care for the patient. SAFE was developed as a collaboration between Oslo University Hospital and three districts in Oslo municipality. The Frogner district initiated the implementation of SAFE to promote systematic use and follow-up of the tool. In addition to identifying early signs of sub acute functional decline in elderly individuals receiving home care services, the initiative also aims to improve the observation skills of employees. This was done by training nurses in how to use SAFE and follow up on the results. The SAFE tool was introduced right after the shutdown in 2020 due to the COVID-19 pandemic. Increased pressure on the municipality's home care service lead to increased pressure on home care, and highlighted a need for better and more holistic assessment tools, which was one of the reasons why SAFE was introduced. SAFE was established as an extension of “Live your whole life,” a quality reform from the government that was introduced in Norwegian municipalities in 2017/2018 and was intended for the home care services. The goal was that all older people should get better help and support to cope with life. It was intended to give training in the use of SAFE and the follow-up of the results. In addition, it was given a medical equipment bag to all executive nurses (Abudayya et al., 2023).

In 2020, a collaboration with VID Specialized University, Faculty of Health Sciences—Institute of Nursing, and the Frogner district of Oslo municipality's home care service was established (Abudayya et al., 2023). The purpose of this collaboration was to gain insight into home care nurses’ perspectives on the use of SAFE. The data collection for this study was conducted one year later, June 2021, which means that the nurses who were interviewed had been using the tool for about a year. It should be taken into account that the tool was introduced in a demanding time.

### Design

In order to address the aims of the study, FG interviews were conducted, transcribed and thematically analyzed. The current qualitative study adheres to the Consolidated Criteria for Reporting Qualitative Research guidelines and employs a qualitative research design (Booth et al., 2014;Willig & Stainton Rogers, 2017).

### Research Question

What are nurses experience with the use and implementation of SAFE, as a tool to make decisions in daily practice in home care services?

### Sample

In this study, the participants were recruited with the assistance of a key person in the municipality who had been involved in introducing SAFE to all employees in the Frogner district. The Frogner home care service is divided into three sections and serves around 750 clients, each of whom is assigned a nurse responsible for coordinating their care.

### Inclusion and Exclusion Criteria

After obtaining permission from the key person, all permanently employed staff nurses working at the Frogner district were asked to participate in this study (*n* = 60), while other staff nursed with temporarily employment status and other health workers were excluded.

#### Data Collection

The study utilized a qualitative research approach to collect data from participants through three FG interviews in the beginning of June 2021 by two interviewers, a moderator (AH) and an observer (AA), with the latter being responsible for recording the interactions. To ensure the quality of the interviews, an experienced co-author (ZP) supervised the first interview. The researchers introduced themselves and the research project before starting the interviews. Each FG interview lasted ∼50 min. A standardized interview guide was used to ensure consistency in data collection (Supplemental File Interview Guide). The FG interviews provided an opportunity for the participants to interact with one another and share detailed and diverse experiences related to the introduction and implementation of SAFE. In qualitative research, the sample size is not predetermined, and the focus is on obtaining rich and varied descriptions of the phenomenon of interest. Data collection in this study continued until data saturation was reached, that is, until nothing new came up or no new information was obtained. The interviews were audio-recorded and securely stored on a research server at VID Specialized University, where the study was conducted. Hermeneutics was used as theoretic framework, and transcribed interviews were analysed using thematic analysis (Clarke et al., 2015). We started from hermeneutics and interpreted the variety and richness of personal experiences that respond to the purpose of the study. The analysis was performed as follows. All co-authors read the transcribed interview text and carried out the analysis independently. When everyone was done, then compared our results. After discussions and consensus, we agreed on the final result.

### Ethical Considerations

The project description, the use of the SAFE instrument, interview guide and informed consent were approved by SIKT-Norwegian Agencie for Shared Services in Education and Research (reference number: 237918). The informed consent to participate in the interviews and the interview guide were sent to all participants prior to the interviews. The participants were informed that the participation was voluntary, and about the right to withdraw from the study at any time without any negative consequences for them. They were informed that the collected data would only be used for the purposes of the present study. The data and personal information have been treated confidentially and in accordance with the General Data Protection Regulations. All data have been anonymized. Only co-authors have access to the transcribed interviews, which are encrypted with a code and stored on the research server. The Key person was not involved in the interviews and had no access to the transcribed anonymized interviews as she has a leading position in the home care service in the Frogner District. Presentation of the results will remain anonymous, and it will not be possible for participants to be identified in publications.

## Results

### Sample Characteristics

A sample of qualified staff (*n* = 16), defined as RNs, agreed to participate in this research, six from Majorstua district, five from Solli district and five from Uranienborg district. The participants’ ages ranged from 25 to 60 years, and both genders were represented (three men and 13 women).

To maintain anonymity, the participants have been anonymized as respondents (R) 1–16 (i.e., R1, R2, R3, …, R16) in the results section of this study. Due to the limited geographic scope of the investigation, no personal information was collected that could reveal the identities of the participants.

### Research Question Results

The data analysis resulted in three themes: (1) Nurses learn how to use SAFE by using it, (2) SAFE's structure promotes patient-centred care through a customized approach to nursing, and (3) SAFE should be integrated into general routines to improve nursing efficiency.

#### Nurses Learn How to Use SAFE by Using It

The participants reported that a short introductory course for SAFE was organized. However, some participants described that SAFE was introduced through direct use of the tool. Several emphasized the lack of practical guidelines to begin proper use of the tool and to clarify issues that arise. There is no general routine for introducing SAFE; it depends mostly on the individual RN and personal initiative. Several participants formulated it like this:

We maybe only had a small explanation of where we were supposed to map the user's functional level and see how it changes by using SAFE (R1).

I haven't been to a course, I started and then I got SAFE on a task with a user, and then I got a sheet, and it was just to work through it … I found a form that said something about SAFE, which I read, but I haven't received any guidance beyond that. No, it's sort of on my own initiative (R2).

Participants reported receiving ongoing support and follow-up from colleagues when using SAFE. Several participants said:

I've worked in several home nursing services in different districts, and then when I started here, I got a bag with various nursing equipment, and the SAFE form was also in the bag. Then on week three of the job, I got an assignment to perform SAFE, so I just asked my colleagues what it was, and that was it (R11).

It would be nice to have something like ..repetition or something that makes you reflect? In other words, a conversation with other nurses and talking around a bit and just refreshing a bit. I guess we take SAFE quite often so that I feel quite confident about that form and so on, getting that conversation going and getting the most out of it since it is of use, it is perhaps a good idea to update SAFE once every six months (R16).

Some participants considered SAFE to be self-explanatory, and several emphasized that it is a good tool for detecting functional decline among older people living at home. Furthermore, it was emphasized that SAFE contributes to systematic mapping and is a good tool for planning work in home care. However, several participants expressed the desire to have general routines for how often SAFE should be used.

… it's good in relation to the clinical aspect, I think. Many people benefit from the results, and you also gain better insight into the patient's needs. You can see the changes, so it's a useful tool … I think it's pretty clear what you need to do with the checklist, I mean, it's about what questions to ask and which measurements to take and things like that (R3).

It might be useful to get some expectations from management about how often we should use that form since it seems like we're doing it very differently and not consistently (R4).

The finding revealed that in order to cover all needs and contribute to a complete assessment of service users, SAFE needs to be developed further by adding additional items regarding different symptoms of physical and psychological functional decline among older people. One participant formulated it in this way:

Because what I sometimes find difficult with that topic is that you end up in the middle. It's not like the person is not in good health, but there are things that (don't) fit perfectly with the condition the person is in, and then you wonder where to write it. So, I always feel the need to add some text such as mental cognitive condition… (R16).

Participants reported that the SAFE project included backpacks equipped with the necessary instruments for the most common examinations, including blood pressure devices, stethoscopes, and thermometers. This was described as a positive boost for daily work. One of the participants said:

Yes, but I'm very proud of the bags. I think it's great that we got a (supply of salt), for example. We had a really bad COPD (chronic obstructive pulmonary disease) patient at that time, and it was really uncomfortable to walk in there if you didn't have one, even though she had her own bag. It's good to have your own equipment on the trip… (R 10).

#### SAFE's Structure Promotes Patient-Centred Care Through a Customized Nursing Approach

The participants of the study described SAFE as a useful tool for preventive work and for facilitating interprofessional collaboration. By structuring a patient's assessment, it allows for proper communication and reporting between different care providers. Participants also mentioned that SAFE helped them identify patients’ needs and involvement and provide prompt referrals to the appropriate healthcare providers. Additionally, RNs and other healthcare assistants can communicate with each other about the results and ask questions. Several participants said:

When I sit with a user and fill out the BASELINE, I try to include the user and do it a bit together, and then the person sees what I write and I feel it becomes a bit of inclusion (R15).

Suddenly, if I find out that someone is very unstable and needs a walker, then I would send a message to the *occupational therapist and get it fixed or have them come for an assessment visit* (R2).

… I also think that both healthcare assistants and regular nurses do it, and if there is something that healthcare assistants are curious about regarding the results (and nurses) … we talk among ourselves, … there is something about communication again, that we talk to each other. If there is something, you just have to ask (R3).

The study highlights the significance of using SAFE regularly and continuously updating it to enhance competence. Knowing the patients well and having continuity in meetings with them, in combination with SAFE, is considered essential for a proper assessment. It is also desirable to have the same person filling out SAFE. While some healthcare workers may know the patients better, others could still take a SAFE assessment and discuss the results with the responsible nurse. Participants emphasized that having the complete support of management is crucial for proper use of SAFE and expressed a desire for more guidance and support in using SAFE effectively, such as through lectures and examples.

Some healthcare workers go more frequently than us, right, they know the users better than us (R6).

I think that [any] healthcare worker could perhaps go and take a SAFE assessment, then they could talk to the service responsible nurse and go through the results [afterwards] (R3).

… yes on Teams! But I don't learn as well from that. I find it easier when a lecturer comes and explains and goes through the points and provides examples. So, it seems to me that it would have been easier to understand what you should write and not write and things like that (R15).

The participants in this study emphasized that the practical use of SAFE involves filling out a paper form, which is then transferred to an electronic data journal called Gerica. However, participants found the process of filling out forms and transferring data to be cumbersome and demanding. RNs also found the filled-out forms in the journal were difficult to understand and time-consuming to navigate. Despite these challenges, participants recognized the importance of using SAFE to gather and share important clinical information for interprofessional collaboration. Participants formulated it like this:

It's a big job because you write down things with a pen at the user's home, and then you have to sit and enter them again on the computer when you get back (R12).

It's just what we enter in the health record that we quickly see if there's been a change in weight since last time or in the past few months, or if the blood pressure has been different, but the actual form (SAFE), we have to work a bit to bring it up (R4).

#### SAFE Should be Integrated into General Routines to Improve Nursing Efficiency

The results of this study highlight the significance of effectively integrating SAFE into daily clinical work and incorporating it into routine tasks. Some participants expressed their wish for SAFE to be available in electronic format directly linked to Gerica. This would allow them to work efficiently with the tool and avoid the duplicative work of manually transferring information from paper forms to electronic devices. Participants recommended digitizing the paper form in Gerica as a time- and effort-saving measure, enabling them to complete the form while with the patient and enter it directly into electronic devices. One of the participants mentioned this:

My wish would be if the form was in Gerica so we could fill it out while we were with the user (in their homes). Then we would have saved both time and work afterwards so we could go in and write it directly into Gerica on the electronic devices (i.e. tablet or mini computer) (R3).

A thorough and accurate completion of the SAFE assessment offers insights into the necessary resources required to address new patient needs. Some participants noted that challenges with registering information in SAFE could discourage its use. Participants also emphasized the need for well-defined standard procedures, including written guidelines on how to utilize completed SAFE assessments. As one participant stated:

It's important that you use it systematically, I mean, you actually have to go in and check the changes or check what was done last if you're going to benefit from it at all (R8).

According to the results of this study, completed SAFE assessments are frequently disposed of or stored in backpacks where they may be exposed to moisture from rain, putting them at risk of damage. The participants acknowledged that using SAFE enhanced their work efficiency and ensures that important observations are not missed. However, there is disagreement among colleagues regarding the frequency and timing of using SAFE, with some colleagues failing to recognize its added value. For many RNs and other healthcare professionals, SAFE is perceived as a project-based task that is not naturally integrated into their daily clinical routines once a project is finished. The participants observed that for some patients there were completed SAFE dating back three years, there were also gaps during which no assessments had been conducted. One participant mentioned:

… I see with some of the users, I can go back three years and find a SAFE (form), and that's because the previous project was done, and then there's a gap where it wasn't taken (R8).

One of the key factors hindering the acceptance of SAFE that participants mentioned was high workload. They expressed that if they received a SAFE form on a particularly busy day, it might not be feasible for them to complete it in Gerica on the same day, and they may have to postpone it for a few days. Despite efforts to facilitate the process, participants still found it challenging to prioritize SAFE assessments with their workload. As a result, many reported relying more on their clinical observations than SAFE. Participants formulated it this way:

So, it's like if you have a SAFE one day, it's completely jam-packed, and it's not guaranteed that you will have time to fill it out in Gerica on that day, maybe you have to do it a few days later… (R7).

… it's not always facilitated for us to take these SAFE even though I know they are trying… (R8).

## Discussion

This study revealed three themes related to the use of the SAFE tool in home care in Norway: (1) Nurses learn to use SAFE by using it. It was perceived by the nurses that there was no general routine for introducing and training in the SAFE tool, but the use was learned through direct use of the tool, (2) SAFE's structure promotes patient-centred care through a customized nursing approach. The nurses described SAFE as a useful tool for preventive work, for facilitating communication between different care providers, and for identifying patients’ needs, and (3) SAFE should be integrated into general routines to improve nursing efficiency. It was expressed a wish for integrating SAFE into daily clinical work and incorporating it into routine tasks, for instance, by incorporating it in the electronic database.

The findings that nurses learn how to use SAFE by using it and that ongoing collegial support is important are consistent with previous research. In a study by Holmström et al. (2020), nurses who used a similar tool in home care reported that they learned how to use it through practical experience and discussions with their colleagues (Holmström et al., 2020). Our study also highlighted the importance of ongoing support and training to maintain competence in using the tool. A study by Söderman et al. (2021) found that nurses who used a nursing documentation system in home care required ongoing support and training to ensure that they could use the system effectively (Söderman et al., 2021). The lack of practical guidelines for proper use of the SAFE tool is a concern, as it may affect the tool's effectiveness in detecting functional decline among older people living at home. Previous research has also highlighted the need for clear guidelines, routines and clinical leadership for using assessment tools in home care (Næss et al., 2023). For example, a study by Oksavik et al. (2020) found that the use of a risk assessment tool in home care was more effective when it was supported by clear guidelines and routines supported (Oksavik et al., 2020). Furthermore, studies have shown that nurses who used a nursing assessment tool in home care reported that it was useful for identifying the needs of older people and improving the quality of care (Zadworna-Cieślak, 2020), and their ability to detect and prevent the deterioration of patients’ health status (Buckley et al., 2016; Gjevjon et al., 2019).

Several studies support the finding that SAFE promotes patient-centred care through a customized nursing approach (Gjevjon et al., 2019; Lindberg et al., 2020; Naess et al., 2017; Ravenswood & Douglas, 2018). For instance, a study by Naess et al. (2017) investigated the implementation of SAFE in home care in Norway and found that the tool promoted patient-centred care by allowing nurses to focus on patients’ individual needs and tailor their care accordingly (Naess et al., 2017). The authors argued that SAFE facilitated interprofessional collaboration, enabling proper communication and reporting between care providers. Other studies explored the use of assessment tools in nursing homes and found that the tool helped identify patients’ needs and aided in prompt referrals to appropriate healthcare providers (De Meester et al., 2013; Gjevjon et al., 2019; Lindberg et al., 2020; Oksavik et al., 2020). This study reported that SAFE facilitated communication among healthcare assistants and other care providers, allowing for more coordinated and efficient care delivery. A study by Bondevik et al. (2017) found that similar assessment tools to SAFE enhanced the quality of care for older people with dementia by promoting a more person-centred approach (Bondevik et al., 2017). SAFE enhanced the quality of care for older people living at home by promoting a more person-centred approach (Gjevjon et al., 2019).

Overall, these studies suggest that SAFE is an effective tool for promoting patient-centred care through a customized nursing approach. The tool enables nurses to identify patients’ needs and tailor their care accordingly, while also facilitating communication and collaboration among different care providers. However, further research is needed to explore the specific mechanisms by which SAFE promotes patient-centred care and to identify the most effective ways of implementing the tool in different care settings.

The integration of SAFE into general routines for nursing efficiency has been recognized as an essential factor in ensuring quality patient care in home care settings. A study by Naess et al. found that incorporating SAFE into daily routines can enhance the quality of care by making it easier to identify patients’ needs and improving communication among healthcare professionals. The authors emphasized the importance of regular updates to the tool to ensure its effectiveness and relevance (Naess et al., 2017). Similarly, a study by Müller et al. (2018) identified the benefits of incorporating standardized tools into general routines for improving the quality of nursing care. The authors found that integrating the SBAR (Situation-Background-Assessment-Recommendation) tool into nursing routines led to more structured communication among healthcare professionals, resulting in better patient outcomes (Müller et al., 2018).

Furthermore, a study by Wagner et al. (2020) emphasized the importance of incorporating structured assessments, such as SAFE, into the general routines of home care services (Wagner et al., 2020). The authors found that incorporating structured assessments into daily routines improved communication and collaboration among healthcare professionals, leading to more efficient and effective care delivery.

Moreover, a regular update of SAFE to remain relevant and effective was recommended by the nurses in this study and is also supported by previous research (Moldskred et al., 2021). Nurses who used a nursing documentation systems and standardized care plans in home care, reported needing regular updates to ensure that the system remained relevant to the needs of patients and the practice of nurses (Larsen et al., 2022; Moldskred et al., 2021; Ree et al., 2019). Overall, the findings of this study highlight the importance of ongoing support and training, clear guidelines and routines, and regular updates to ensure the effectiveness of tools in home care.

## Strengths and Limitations

The strengths of this study are that it provides valuable insights into staff nurses’ experiences using SAFE in home care nursing. These insights provide valuable information and knowledge about how to further facilitate the implementation of SAFE. The sample was diverse, drawn from the entire district's home care nursing staff. This study has some limitations. Firstly, the participants mainly came from one district within a municipality, primarily affiliated with a large home care service. This could limit the study's geographic scope. Additionally, the study was conducted during a pandemic, which could have influenced the participants’ experiences due to resource constraints and work pressure. Secondly, the purposive sampling method could limit the study's generalizability to the wider population of home care nurses.

## Implication for Practise

Use of SAFE as an assessment tool for all new users of home care services, and as a tool to follow-up the users, aims to increase the quality of home care. SAFE helps nurses to detect changes in health and physical function in users of home care services and gives the nurses a potential to initiate nursing measures at an early phase of disease. It is a way to systematically and continually follow-up the users of health care services and to prevent serious decline in health of frail older people.

## Conclusion

Overall, the findings of this study highlight the importance of using tools such as SAFE in home care settings to promote patient-centered care, improve nursing efficiency, and enhance the quality of care delivered in the clinic. The study suggests that thorough introduction and training in the use of SAFE is important for the nurses who are going to take the tool in use in their clinical practise. Furthermore, ongoing support, training and clear guidelines were perceived as necessary.

Finally, incorporating SAFE into general routines has been recognized as an essential factor in improving the quality of care in home care settings. Regular updates to the tool and ongoing support from colleagues can enhance its effectiveness and ensure that patients’ needs are adequately addressed. The use of structured assessments and standardized tools in general routines can improve communication and collaboration among healthcare professionals, resulting in better patient outcomes. The study's findings are consistent with previous research and can enhance the development and implementation of tools in home care, improve patient outcomes and the quality of care delivered.

## Supplemental Material

sj-png-1-son-10.1177_23779608231187246 - Supplemental material for Home Care Nurses’ Experiences of the Use and Introduction of the Subacute Functional Decline in the Elderly Instrument Click here for additional data file. Supplemental material, sj-png-1-son-10.1177_23779608231187246 for Home Care Nurses’ Experiences of the Use and Introduction of the Subacute Functional Decline in the Elderly Instrument by Nima Wesseltoft-Rao, Zada Pajalic, Annabel Hamre and Abdallah Abudayya in SAGE Open Nursing
