# Supplementary figures and images for: Home Care Nurses’ Experiences of the Use and Introduction of the Subacute Functional Decline in the Elderly Instrument
Source: SAGE Open Nurs. 2023 Aug 10;9:23779608231187246. doi: 10.1177/23779608231187246 (PMC10413904; doi:10.1177/23779608231187246)

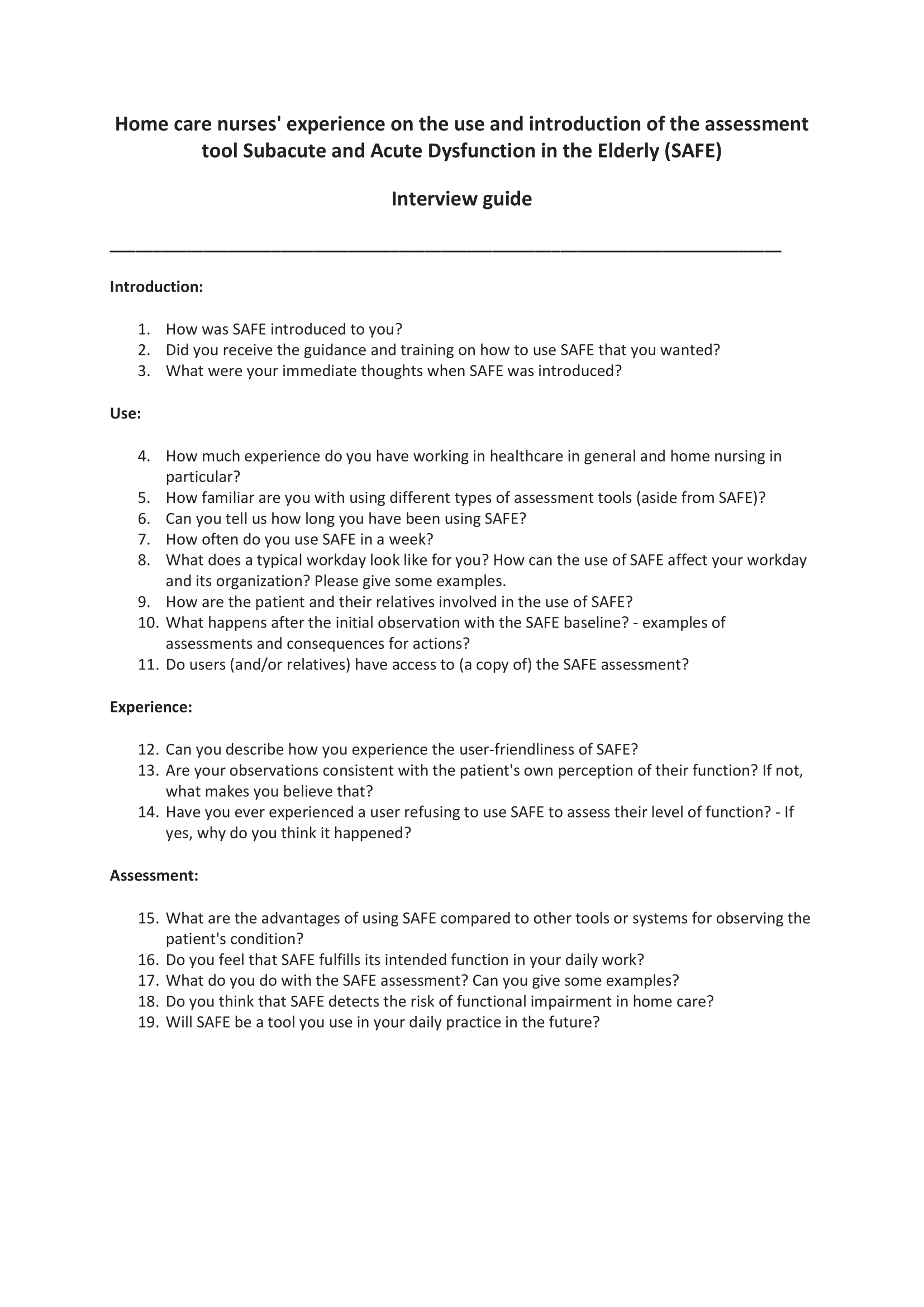

Supplement: sj-png-1-son-10.1177_23779608231187246 - Supplemental material for Home Care Nurses’ Experiences of the Use and Introduction of the Subacute Functional Decline in the Elderly Instrument [file sj-png-1-son-10.1177_23779608231187246.png]
